# Neonatal enteral antibiotics reduce gut inflammation and delay systemic immune development in preterm pigs

**DOI:** 10.1038/s41390-025-04436-9

**Published:** 2025-10-03

**Authors:** René Liang Shen, Ziyuan Wu, Xiaoyu Pan, Shuqiang Ren, Anders Brunse, Per Torp Sangild, Duc Ninh Nguyen

**Affiliations:** Comparative Pediatrics, Department of Veterinary and Animal Sciences, Faculty of Health and Medical Sciences, University of Copenhagen, Copenhagen, Denmark

## Abstract

**Background:**

Antibiotics are frequently administered to preterm infants after birth to prevent or treat severe infections. However, interactions between gastrointestinal health and systemic immune development following neonatal antibiotics are unclear.

**Methods:**

Using a preterm pig model, we investigated the systemic immune effects of four days of antibiotics (AB) treatment just after birth. Preterm pigs received enteral AB for 4 days and were compared with controls (water) until 9 days after birth (*n* = 28–32). Blood samples were collected at birth and on days 5, 7, and 9. Gut samples were collected on day 9.

**Results:**

Expression of TLR2, TLR3, S100A9, and IL10 differed by day 5, 7 and 9 in blood for controls, while AB-treated showed delay of these temporal developments. On day 9, blood transcriptomics revealed 1765 DEGs (1090 downregulated) in AB-treated pigs compared with controls, with suppression of inflammatory and energy metabolism pathways. The suppression was associated with lower intestinal permeability, bacterial adhesion and gut inflammation. The findings suggest that enteral AB exposure after preterm birth, reduces not only gut inflammation, but also delay systemic immune cell development.

**Conclusion:**

Neonatal AB treatment might reduce gut inflammation; however, it may cause subsequent reduced capacity to combat systemic infections.

**Impact statement:**

- Neonatal antibiotics attenuate gut inflammation and systemic immune development.
- Effects persist after cessation of antibiotic treatment.
- Inflammatory pathways in blood are associated with gut health parameters.
- This study underscores the complex interplay between gastrointestinal health and systemic immune function.
- Neonatal antibiotics may influence risk of infectious complications even after treatment.

## Introduction

Preterm infants, defined as those born before 37 weeks of gestation, are particularly vulnerable to a range of morbidities, including neonatal infection and inflammation.^1^ Early-life antibiotic therapy is essential for managing bacterial infections and represents the most frequently administered medication in neonatal intensive care units (NICUs).^2^ Administration of antibiotics to very low birth weight (VLBW) infants within the first days of life occurs for 50–100% of infants, with a duration ranging from a few days to 2 weeks.^3^ However, the widespread and prolonged use of antibiotics in neonatology carries significant risks, including antimicrobial resistance, disruptions in immune system development, and an elevated risk of subsequent infections.^4^ Clinical data has demonstrated a link between prolonged antibiotic exposure and critical outcomes, such as increased mortality, late-onset sepsis, and necrotizing enterocolitis (NEC).^5,6^ These findings underscore the urgent need for judicious antibiotic use in NICUs to balance the benefits of infection control with the prevention of adverse short- and long-term effects.

NEC is a life-threatening gastrointestinal disorder affecting 5–10% of preterm infants with very low birth weight (VLBW, <1500 g).^7–9^ The disease is marked by profound intestinal inflammation and bacterial invasion of the bowel wall and predominantly affects formula-fed neonates.^7–9^ While broad-spectrum antibiotics play a critical role in the treatment of NEC, their role in preventing the condition remains contentious. Retrospective studies have yielded conflicting results, and some suggest that early antibiotic use increases the risk of NEC, while others point to a protective effect.^10^ Moreover, prolonged or excessive antibiotic treatment can result in significant gut dysbiosis, disrupting the microbial balance and potentially impairing intestinal immunity.^11–15^ Regardless, direct or indirect links among early gut development, neonatal antibiotics, and later systemic immune responses are not fully understood.

Preterm pigs delivered by cesarean section at 90% gestation are a valuable model for studying systemic immune development in very preterm infants due to many shared phenotypic characteristics, including low blood leucocyte counts, underdeveloped mucosal and blood bacterial defense mechanisms, impaired responses to infectious challenges,^16,17^ together with general immaturity of multiple organs (gut, liver, brain, liver, kidney).^18–20^ Preterm pigs are highly susceptible to NEC, especially during the first week of life and when fed formula, reflecting the diet-, immune-, and microbiota-dependent nature of NEC in the first few weeks in preterm infants.^21^ Our previous research demonstrated that neonatal AB treatment markedly reduced NEC sensitivity and altered microbiota composition, but recolonization occurred rapidly after discontinuation of therapy.^21–23^ A different study showed that fecal microbiota transplantation (FMT) had limited effects on gut re-colonization, mucosal structure/function, or systemic immune parameters on day 9 after discontinuing AB treatment after 4 days.^23^ Building on this previous work (using the same animals), we hypothesized that a 4-day regimen of neonatal AB treatment would reduce intestinal inflammation throughout 9 days but also modulate (directly or indirectly) systemic immune and inflammatory responses. Hence, this study aimed to understand further how early-life AB affects later systemic immune development and also assess associations between these immune responses and gut parameters.

## Methods

### Animal procedures and antibiotic treatments

The Danish Animal Experiments Inspectorate approved all animal studies and experimental procedures under license number 2020-15-0201-00520. These approvals are in accordance with EU Directive 2010/63, which governs the legislation for the use of animals in research. Sixty-seven crossbred piglets (Landrace x Large White x Duroc) were delivered via cesarean section at day 106 (90% gestation, term at day 117) from three healthy sows, following established methods.^23^ Immediately post-delivery, the newborns were placed in individually ventilated incubators preheated to 37 °C and supplied with an initial oxygen flow rate of 1-2 liters per minute. Subsequently, animals were fitted with an umbilical arterial catheter (4 F; Portex, Kent, UK) for parenteral nutrition and blood sampling and an orogastric feeding tube (6 F Portex) for enteral nutrition. Then, the pigs were categorized based on sex and birth weight and randomly assigned to groups receiving oral administration of antibiotics (AB, *n* = 28) or sterile water (CON, *n* = 32). The enteral antibiotic regimen consisting of amoxicillin (50 mg/kg/day) and clavulanic acid (12.5 mg/kg/day), combined with neomycin (50 mg/kg/day), administered twice daily during the first four days of life. The selection of antibiotic drugs and administration routes were based on previous studies on the effects of enteral antibiotics on gut parameters and considerations of commonly used antibiotics in pediatrics and also veterinary medicine.^21,24–26^

Between postnatal days 1 and 9, all animals were provided with specialized formula milk modified for preterm pigs, administered by an orogastric tube every three hours. The feeding regimen included a progressive increase in volume, from 16 to 112 ml/kg/day, accompanied by a proportional reduction in parenteral nutrition support, which decreased from 144 to 48 ml/kg/day (Fresenius Kabi, Denmark). The details of the formula composition are shown in Table S2. From days 5 to 7, a subset of animals from each group (CON-FMT, *n* = 17 and AB-FMT, *n* = 16) received a rectal administration of donor feces, constituting fecal microbiota transplantation (FMT) sourced from a healthy, full-term pig, as part of a 2 × 2 study design.^23^ As described previously, the FMT treatment did not influence clinical or hematological parameters, thus directing the current study’s primary focus toward evaluating the specific temporal and developmental impacts of neonatal antibiotic administration. Data from all four groups are available in Supplementary Materials, but for this report, the pooled data for all AB and non-AB pigs are presented.

On day 9, five days after the 4-day AB treatment, animals were anesthetized and subsequently euthanized via intracardiac barbiturate injection. The procedures for necropsy and the subsequent collection and analysis of gastrointestinal parameters (lactulose-mannitol ratio, CD3 area fraction, mucin area fraction, MPO staining, FISH staining, brush border enzyme activites, tissue cytokine levels), including gut microbiota assessment, were conducted as previously described.^23^ Blood samples obtained from the umbilical arterial catheter on days 5, 7, and 9 were subjected to hematological analysis using the ADVIA 2120i Hematology System (Siemens, Germany). Additional blood samples collected on days 1, 5, 7, and 9 were used for quantitative PCR (qPCR), with the rest of the samples on day 9 allocated for whole blood transcriptomics analysis.

### Temporal gene expression using qPCR

Total RNA was extracted from whole blood samples collected longitudinally on days 5, 7, and 9 using the MagMax 96 blood RNA isolation kit, following the protocol previously established.^27^ The qPCR was employed to measure the relative mRNA expression levels of 22 preselected genes associated with various immune functions and metabolic processes. These expression values were normalized to the reference gene, HPRT1, to ensure accurate quantification. Primer sequences were carefully designed using the Primer-BLAST tool from the National Institutes of Health (Bethesda, Maryland), with all sequences cataloged in Supplemental Table S3 for reference.

### Whole blood transcriptomics

Blood samples collected on day 9 were randomly selected from each experimental group (AB, *n* = 8; CON, *n* = 10) for transcriptomic analysis. Gene expression profiling was conducted using whole-transcriptome shotgun sequencing. Library preparation and sequencing were carried out by NOVOGENE (Cambridge, UK). Specifically, RNA-Seq libraries were constructed using 1000 ng of RNA and the VAHTS mRNA-Seq V3 Library Prep Kit for Illumina (Vazyme, Nanjing, PRC). Sequencing was performed on the Illumina NovaSeq 6000 platform, yielding 150 bp paired-end reads. Quality control, including trimming of adapters and low-quality bases, was conducted using TrimGalore (Babraham Bioinformatics, Cambridge, UK). The resulting clean reads, with an average of approximately 26 million per sample, were aligned to the porcine genome (Sscrofa11.1) using Tophat2.^28^ Gene annotation data for the porcine genome were retrieved from *Ensembl* (release 99), and the *htseq-count script*^29^ was used to generate a gene-count matrix.

To identify differentially expressed genes (DEGs), we first excluded genes with low expression levels. The *DESeq2* package (version 1.38.3)^30^ was employed to perform DEG analysis between the AB and CON groups, with litter as the covariate in the model. Robust fold-change (FC) estimations were obtained using the *lfcShrink* function with *ashr*, applying a false discovery rate (FDR) threshold of 0.05.^31^ Gene set enrichment analysis (GSEA) was performed via the *clusterProfiler* package (version 4.0.2)^32^ to identify significantly affected pathways. Genes were ranked based on log-transformed FC values derived from DEG analysis before pathway assessment. The Kyoto Encyclopedia of Genes and Genomes (KEGG) database for *Sus scrofa* was utilized, and an FDR cut-off of 0.05 was applied to determine pathway significance. Normalized enrichment scores (NES) were calculated, where negative and positive values indicated down- and up-regulated pathways, respectively. Pathway-enriched gene expression was visualized using heatmaps generated with the *ComplexHeatmap* package (version 2.15.1).^33^ Gene set variation analysis (GSVA) was conducted utilizing the *GSVA* and *GSEABase* packages to evaluate pathway-level expression changes.^34^ Scores generated through GSVA were subsequently used to analyze differential expression via the *limma* package.^35^ The KEGG database for *Sus Scrofa* served as a reference for identifying relevant pathways. Significantly upregulated and downregulated pathways were determined based on FDR  <  0.05 and |log2FC | > 0.4. Additionally, GSVA scores calculated across all samples were employed in Spearman’s correlation analyses to explore associations with clinical parameters, providing insights into the potential relationship between gastrointestinal parameters and identified pathways.

### Statistical analyses

Statistical analyses were conducted using R software (version 4.2.3). Continuous variables were examined via linear mixed models, with temporal effects assessed as repeated measurements within a longitudinal data framework, incorporating subjects as random effects, using the *lmer* package.^36^ This allowed for comparisons of changes over time relative to baseline values established on day 1. A Tukey adjustment was applied to correct for multiple comparisons within longitudinal models using the *multcomp* package.^36^ Linear mixed models included adjustment for litter, sex, birth weight, and FMT intervention. Logarithmic transformations were applied to improve normality for data not conforming to a normal distribution. In cases where normality assumptions remained unmet, non-parametric methods, specifically the Kruskal-Wallis and Mann-Whitney U tests, were employed to assess statistical significance. Statistical significance was defined as a *P* value < 0.05. Detailed outcomes for all four experimental groups, along with statistical analyses showing the overall effects of AB treatment, FMT treatment, and their interaction, are presented in Table S4.

## Results

### Broad-spectrum antibiotic treatment reduces gut inflammation and delayed development of immune cell profiles

Briefly, as presented in the separate paper,^23^ following four days of AB treatment, animals in the AB group demonstrated a higher daily weight gain than those in the CON group, but this effect was less pronounced from days 5 to 9 (after AB treatment ceased, Table S1). While no differences were detected in NEC scores or in the duration of diarrhea episodes, AB treatment notably reduced gut permeability. For parameters associated with the small intestine, AB treatment led to decreases in CD3-stained area (indicative of T cell density), bacterial FISH-stained area (reflecting bacterial adhesion), and MPO-stained area (a measure of macrophage and neutrophil granulocyte density), without affecting mucin density. Enzymatic analysis showed that AB treatment increased brush border peptidase activity, particularly for enzymes ApN and ApA. Additionally, there was a tendency for decreased levels of the anti-inflammatory cytokine IL-10 in AB-treated animals. In the colon, mucin density and MPO-stained area were elevated in the AB-treated animals, with no significant changes observed in CD3- or FISH-stained areas (with high variability). Finally, levels of the pro-inflammatory cytokine TNF-α and counts of anaerobic bacteria were reduced in the colon of AB-treated animals. More details are available in a separate paper.^23^

On day 5, AB-treated animals demonstrated higher levels of various immune cells in the blood, including white blood cells, lymphocytes, and neutrophils, with the exception of monocytes, which were observed at lower levels compared to CON animals (Fig. S1A–D). By day 7, while white blood cells, lymphocytes, and neutrophils showed no significant difference between AB-treated and CON animals, monocyte and platelet counts remained lower in the AB-treated group (Fig. S1A–D). On day 9, in contrast to the elevated cell counts in the CON group, AB-treated animals exhibited significantly lower levels of white blood cells, lymphocytes, neutrophils, monocytes, and platelets (Fig. S1A–E). Throughout days 5 to 9, red blood cell counts did not differ significantly between AB-treated and CON groups (Fig. S1F).

### Broad-spectrum antibiotic treatment reduces the expression of immune and inflammatory genes in the blood

**Fig. 1:**
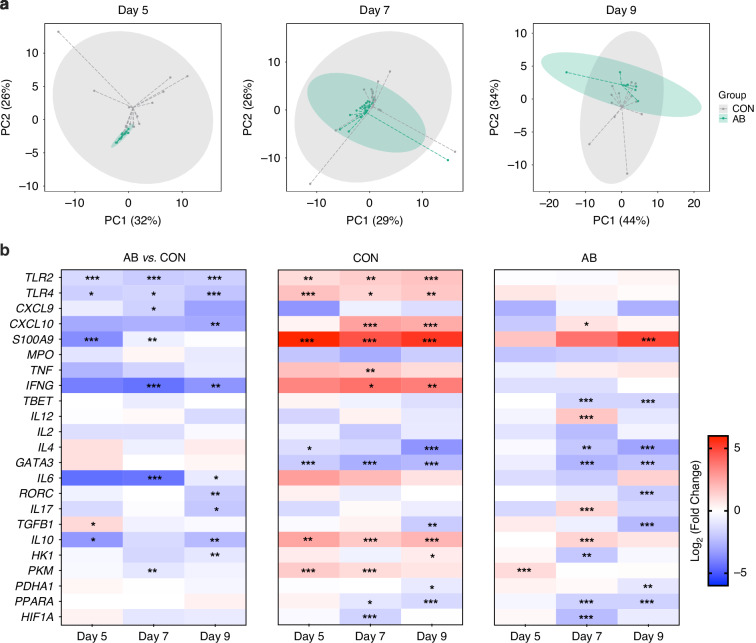
Broad-spectrum antibiotic treatment reduces expression of immune and inflammatory genes in the blood. **a** PCA scores plot of the first two principal components from relative expressions of 23 genes by qPCR. The colored ellipses represent 95% confidence intervals for each group. **b, left** Whole blood gene expressions related to immune functions on day 5, 7, and 9 between AB and CON animals are shown as relative fold changes in relation to HPRT1 expression. **b, middle & right** Whole blood gene expressions related to immune functions on day 5, 7, and 9 in AB and CON groups, respectively, are shown as relative fold changes in relation to expression on day 1. The heatmap color palette represents the lowest fold change with cool colors, while warm colors represent the highest values. CON, control; AB, antibiotics; HPRT1, hypoxanthine phosphoribosyltransferase. Relative gene expression in the whole blood by reverse transcription quantitative real-time PCR. ^∗^*P* < 0.05, ^∗∗^*P* < 0.01, ^∗∗∗^*P* < 0.001. TLR2 Toll-like receptor 2, TLR4 Toll-like receptor 4, CXCL9 C-X-C motif chemokine ligand 9, CXCL10 C-X-C motif chemokine ligand 10, S100A9 S100 calcium-binding protein A9, MPO myeloperoxidase, TNF tumor necrosis factor, IFNG interferon gamma, TBET T-box expressed in T cells, IL12 interleukin 12, IL2 interleukin 2, IL4 interleukin 4, GATA3 GATA-binding protein 3, IL6 interleukin 6, RORC RAR-related orphan receptor C, IL17 interleukin 17, TGFB1 transforming growth factor beta 1, IL10 interleukin 10, HK1 hexokinase 1, PKM pyruvate kinase M1/2, PDHA1 pyruvate dehydrogenase E1 subunit alpha 1, PPARA peroxisome proliferator-activated receptor alpha, HIF1A hypoxia-inducible factor 1-alpha.

Given the substantial effects of AB treatment on clinical parameters and systemic immune cell profiles, we employed qPCR to quantify 23 genes in blood, examining differential responses in innate and adaptive immune functions, inflammation, and metabolic activity between the AB and CON groups on days 5, 7, and 9. Principal component analysis (PCA) demonstrated a progressively greater separation between AB-treated and CON animals from day 5 through day 9 (Fig. 1a). In comparison with CON animals, the AB group showed reduced expression of innate immune and chemokine-related genes, including *TLR2*, *TLR4*, *CXCL9*, *CXCL10*, and *S100A9* (Fig. 1b, left and Fig. S2A–D), with *S100A9* expression reduction proving transient, as marked differences on day 5 were no longer significant by day 9. Genes associated with adaptive immune responses, including *IFNG*, *IL-6*, *IL-10*, *IL-17*, and *RORC*, also showed lower expression in AB-treated animals, particularly on day 9, except for *TGFB1* on day 5. Energy metabolism genes *HK1* and *PKM* displayed decreased expression on days 5 and 7, respectively. The temporal analysis found that CON animals displayed an upward trajectory in gene expression from day 5 to day 9 vs. day 1, including genes for innate immune response (*TLR2*, *TLR4*, *CXCL10*, and *S100A9*), adaptive immunity (*TNF*, *IFNG*, and *IL10*), and metabolism (*HK1* and *PKM*) (Fig. 1b, middle). In contrast, AB-treated animals exhibited very limited temporal changes in innate immune and chemokine-related genes, except for *S100A9*, which showed a delayed increase that only became significant by day 9 (Fig. 1b, right). AB animals also demonstrated reduced expression in adaptive immune response genes, such as *TBET*, *IL4*, *GATA3*, *RORC*, and *TGFB1*, over days 5 to 9 compared with day 1, though *IL-10*, *IL-12*, and *IL-17* were exceptions with elevated expressions. Additionally, *HK1* expression in AB animals decreased on day 7 relative to day 1.

### Whole blood transcriptome to confirm and explore the effects of antibiotics

**Fig. 2:**
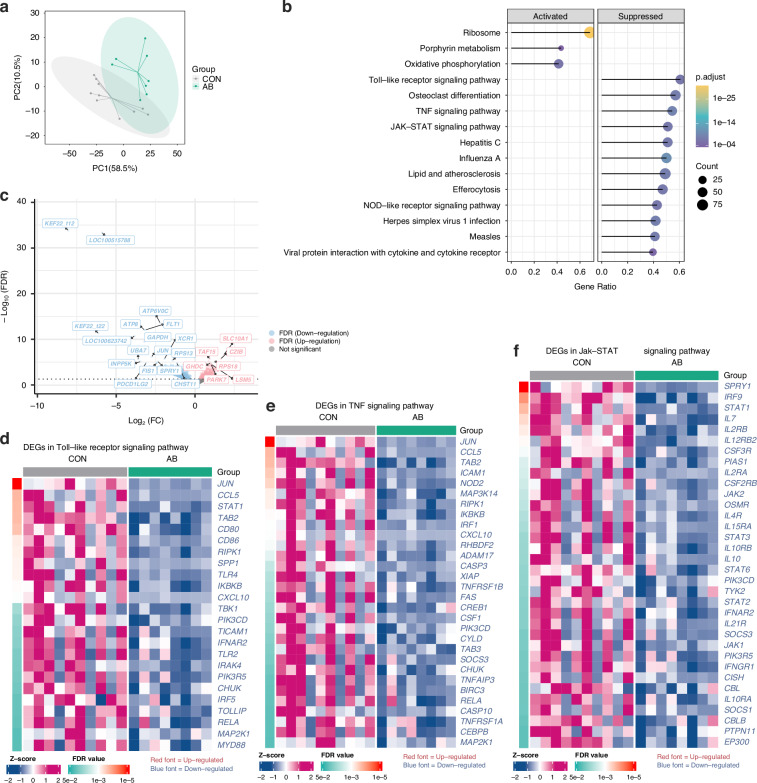
Whole blood transcriptome to confirm the effects of broad-spectrum antibiotics on immune and inflammation genes. **a** Whole blood Transcriptomics PCA scores plot of the first two principal components. The colored ellipses represent 95% confidence intervals for each group. **b** GSEA was performed between the AB and CON groups using the *Sus scrofa* (pig) KEGG knowledgebases. The top significant pathways ranked by FDR value have been selected for visualization. The size and color of the dots indicate the gene ratio and FDR values, respectively. The complete list of enriched pathways can be found in Table S3. **c** Volcano plots between AB and CON group. A threshold of FDR < 0.05 was set to illustrate the differentially expressed genes (DEGs). **d**–**f** Heatmap illustrating DEGs involved in the Toll-like receptor, TNF, and JAK-STAT signaling pathways between AB and CON groups. CON control, AB antibiotics, GSEA gene set enrichment analysis.

To further explore blood gene expression patterns, we conducted a comparative analysis of global blood gene expression profiles using bulk RNA sequencing (RNA-seq) on day 9. Principal component analysis (PCA) demonstrated distinct clustering of AB and CON groups, suggesting a marked impact of AB treatment on blood gene expression (Fig. 2a). Gene set enrichment analysis (GSEA) further confirmed that immune and inflammation-related pathways were suppressed in AB-treated animals, including Toll-like receptor, TNF, and JAK-STAT signaling pathways (Fig. 2b and Table S5). In contrast, ribosome function, porphyrin metabolism, and oxidative phosphorylation pathways were notably upregulated in AB-treated animals. Differential gene expression analysis revealed that 675 genes were upregulated while 1090 were downregulated in AB-treated animals compared to CON animals (Fig. 2c). Most notably, genes within immune and inflammation-related pathways were predominantly downregulated in AB-treated animals, as seen by the Toll-like receptor (24 downregulated/0 upregulated), TNF (30 down/0 up), and JAK-STAT (34 down/0 up) pathways (Fig. 2d–f).

### Blood immune gene expressions are associated with gut parameters

**Fig. 3:**
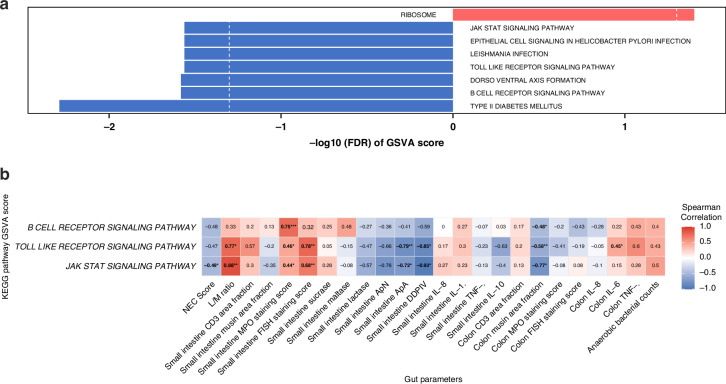
Blood immune pathways are associated with gut parameters. **a** GSVA was performed between the AB and CON groups using the *Sus scrofa* (pig) KEGG knowledgebases. **b** Heatmap representation of Spearman’s correlation analyses between the GSVA scores from all animals of the three signaling pathways and the clinical parameters in Supplementary Material (Table S1). The heatmap color palette represents the lowest correlation coefficient values with cool colors, while warm colors represent the highest values. ^∗^*P* < 0.05, ^∗∗^*P* < 0.01, ^∗∗∗^*P* < 0.001. CON control, AB antibiotics, GSVA gene set variation analysis.

**Fig. 4:**
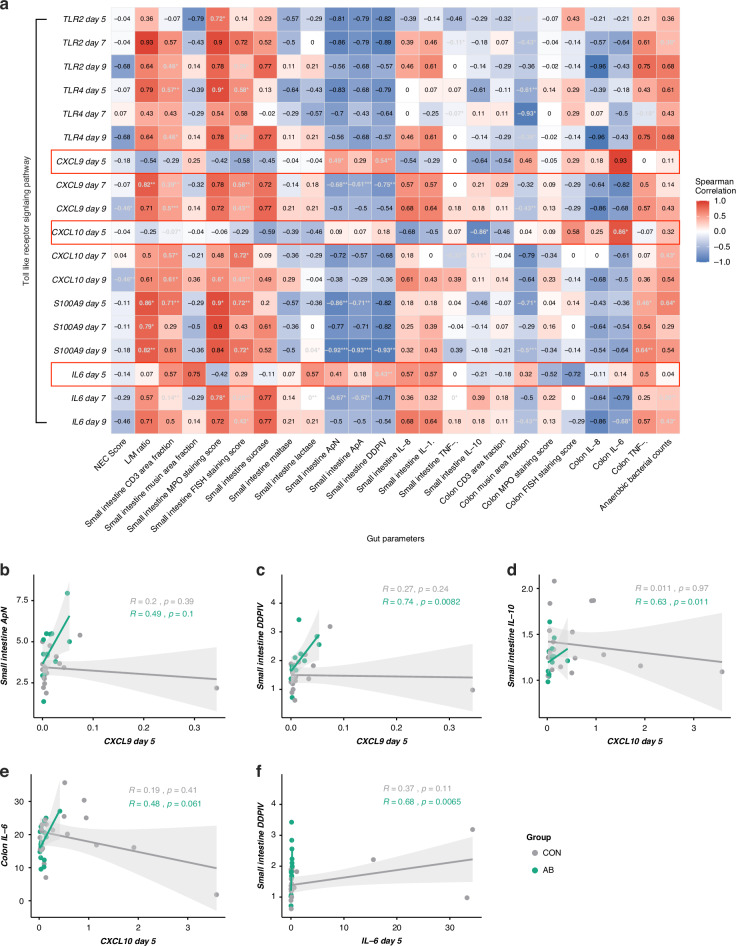
Blood immune genes are associated with gut parameters. **a** Heatmap representation of Spearman’s correlation analyses between clinical parameters and qPCR-measured gene expressions related to the Toll-like receptor and JAK-STAT pathways from days 5 to 9. The heatmap color palette represents the lowest correlation coefficient values with cool colors, while warm colors represent the highest values. **b–f** Spearman’s correlation analyses between clinical parameters and *CXCL9*, *CXCL10*, and *IL-6* expressions on day 5 in AB and CON groups, separately. ^∗^*P* < 0.05, ^∗∗^*P* < 0.01, ^∗∗∗^*P* < 0.001. CON control, AB antibiotics.

The gene set variation analysis (GSVA) was employed to evaluate critical pathways that could potentially show links to gut conditions. Results revealed one pathway showing significant upregulation, while seven pathways showed significant downregulation, including the B cell receptor, Toll-like receptor, and JAK-STAT signaling pathways (Fig. 3a). To link these pathway changes with gastrointestinal conditions, Spearman’s correlation analyses were conducted between the GSVA scores of the three signaling pathways and the clinical parameters in Table S1. Results revealed Toll-like receptor and JAK-STAT pathways had strong positive correlations with several indicators of severe gastrointestinal impairment, including gut permeability (lactulose/mannitol, L/M ratio), macrophage and neutrophil granulocyte densities (as shown by small intestine MPO staining scores), bacterial adhesion (small intestine FISH staining scores), and inflammatory response (colon IL-6 levels) (Fig. 3b and Table S6). Conversely, negative correlations were observed between these signaling pathways and parameters of gut damage, including NEC score, brush border peptidase activities (ApA and DPPIV), and colon mucin density. At the gene level, we further investigated correlations between clinical parameters and qPCR-measured gene expressions related to the Toll-like receptor and JAK-STAT pathways from days 5 to 9 (Fig. 4a). After the AB treatment period, similar correlation profiles among six genes were observed, showing positive correlations with parameters of gastrointestinal impairment, including gut permeability, densities of T cells, macrophages, and neutrophil granulocytes, as well as bacterial adhesion and inflammation. In contrast, these genes were negatively correlated with brush border peptidase activity and colon mucin density. These findings support the above pathway association results. Notably, expression levels of *CXCL9*, *CXCL10*, and *IL6* on day 5 exhibited an inverse pattern compared with days 7 and 9, showing negative correlations with brush border peptidase activities (ApA and DDPIV) and an inflammation marker (small intestine IL-10 response), while being positively correlated with colon IL-6 response. Spearman’s correlation analyses further examine the differential gene responses between AB and CON animals. Notably, while AB animals demonstrated lower overall expression levels of these three genes compared with CON, the AB treated showed a strong positive correlation with parameters indicative of improved gastrointestinal health, whereas this correlation was not seen in CON animals (Fig. 4b–f).

## Discussion

Antibiotic treatment in NICUs is widely used for managing suspected bacterial infections just after birth, yet its effects on the interplay between systemic and gut immune development remain poorly understood. In this study, we investigated the effects of a four-day neonatal antibiotic regimen and found that by day 9 post-treatment, this treatment suppressed blood immune cell profiles and downregulated immune and inflammatory gene expression. Previous work have found comparable immune suppression as signs of immunological immaturity (Nguyen et al.^17^ Sci Rep) (Bæk et al. 2020 Front Immunol). In this study, the dampened systemic immune phenotype was positively associated with improved gastrointestinal indicators, suggesting a complex interaction between systemic immune modulation and gut health outcomes. The reduced immune response in AB animals could be (directly or indirectly) linked to the effects of AB on gut parameters. These findings underline the need for further research to unravel the mechanistic time- and age-dependent pathways that link systemic immune development with gut parameters, including effects of antibiotics, to carefully balance the need to defend the gut and blood against harmful infections and inflammation and at the same time avoid prolonged immune suppression with later risks of infections.

In our earlier studies on short-term antibiotics treatment (4 days), we observed very marked improvements in gut health parameters on day 5.^23^ In this study, animals receiving AB treatment showed improved daily weight gain mainly from day 1-5 during AB treatment, whereas days after cessation of AB on day 9, several intestinal parameters were better compared with CON (i.e. Reduced gut permeability, less tissue damage, lowered anaerobic bacterial burden and adhesion, decreased immune cell densities, suppressed pro-inflammatory cytokine responses). The specific regional and temporal effects appear complex as colon conditions were worse in the AB treated. Systemically, AB-treated animals demonstrated sustained suppression of immune cell profiles, including white blood cells, lymphocytes, neutrophils, and monocytes, following broad-spectrum antibiotic administration. Blood gene qPCR analysis revealed significant downregulation of genes associated with both innate and adaptive immune responses in AB-treated animals post-treatment. While other genes related to immune responses displayed prolonged upregulation post-treatment in CON animals, AB-treated animals maintained consistently lower expression levels, potentially due to reduced gut inflammation induced by the antibiotic regimen. The gene *S100A9*, crucial for intestinal immunity and microbial colonization during early postnatal development,^37^ remained with low expression on days 5 and 7 compared with CON but was notably upregulated on day 9, five days after the cessation of AB treatment. This temporal expression pattern aligned with findings of reduced bacterial burden on day 9, suggesting a transient suppression of gut bacterial colonization both during and after antibiotic intervention.

PCA of the blood transcriptome demonstrated distinct effects of AB treatment, with pathway enrichment analysis highlighting the impact on multiple immune pathways, such as TLR, TNF, JAK-STAT, and NOD-like receptor signaling, following AB discontinuation. Heatmap visualization corroborates these findings by confirming the downregulation of specific DEGs within these pathways. Further analysis with GSVA revealed strong positive correlations between Toll-like receptor and JAK-STAT pathway activity and markers of gastrointestinal impairment. TLRs, known for recognizing microbial patterns (MAMPs) from gut microbiota and activating immune responses, reflect alterations in gut microbiota composition or intestinal barrier function when their expression in blood cells is dysregulated.^38,39^ Similarly, JAK-STAT signaling, which governs immune cell functions, underscores ongoing inflammatory activity in the gut when modulated in blood cells.^40^ Correlation analysis of nine TLR pathway genes showed similar profiles among six genes, which were positively associated with gastrointestinal impairment markers. Interestingly, expression of three TLR-related genes (*CXCL9*, *CXCL10*, and *IL6*) on day 5 displayed inverse trends compared to days 7 and 9, with reduced expression in AB-treated animals positively correlating with improved gastrointestinal health metrics. *CXCL9* and *CXCL10*, key mediators recruiting CXCR3+ immune cells to inflamed gut tissue, are markedly elevated in both experimental colitis models and IBD patients.^41,42^ These findings illustrate the potential and complexity of circulating mRNA/qPCR markers to predict or monitor intestinal conditions, as found by others.^43–47^

The suppressed blood immune responses in AB-treated animals on day 5 may play a role in gastrointestinal health by day 9. Others have described the duality of antibiotic effects on the gut microbiota and host immunity, finding reduced levels of IFN and IL17, consistent with our findings. It has also been shown in germ-free mice that antibiotics can modulate immune metabolism independent of the microbiome.^13,48^ A temporal correlation between gut health and blood immunity may contribute to resolving or mitigating gastrointestinal disturbances. Antibiotic exposure during critical postnatal periods disrupts the natural trajectory of immune system maturation and gut microbiota establishment, potentially creating vulnerabilities. Such disruptions could predispose individuals to increased infection risks once antibiotic treatment ceases, as the balance between immune regulation and microbial colonization may be compromised, highlighting a need for further investigation into the duration and implications of these effects. Retrospective studies with preterm infants find increased LOS after longer duration antibiotic treatment ( >5 days) while the effect of shorter duration antibiotics is limited, and two small randomized controlled trials in preterm infants found no differences in infection outcomes after treatment with 2 days of antibiotics treatment.^5,6,49–52^

Although this study yielded significant insights into the effects of a four-day AB treatment administered at birth, several limitations must be considered. Firstly, the study primarily assessed outcomes by day 9, limiting the understanding of how variations in the timing and duration of AB treatment might influence postnatal development and immune responses. Future studies should explore alternative regimens, examining both earlier and later treatment schedules as well as extended or shortened durations, to fully characterize the therapeutic and adverse effects. Gut tissue analyses from the earlier phases during AB treatment (days 5 and 7) could have provided valuable information but were not possible to collect with this study design. Second, the findings may depend on AB treatment regimens, i.e., type, dose, route, duration, and patient age, as well as nutrition and feeding protocol. The antibiotic regimen used in the study differs from what is used as standards for infants. Preterm infants are treated with antibiotics intravenously rather than enterally, it is possible that intestinal effects differ for route of antibiotic administration. Previous work in pigs found limited protective effects of intravenous antibiotics against NEC compared with enteral treatment (Birck et al. ^21^ AJP). Then, the effects on immunity may also differ with route of antibiotic treatment. Furthermore, although the previous study found limited effects of FMT on hematologic outcomes, potential influence of this intervention may be present. The current study does not investigate potential interaction effects, rather focuses on main effects of the AB intervention. Lastly, the limited volume of blood samples in our study precluded transcriptome analyses on days 5 and 7, which could have provided valuable insights into the dynamics of the host immune response during the post-treatment period.

## Conclusion

This study shows that administering a four-day course of enteral AB treatment immediately after birth in preterm formula-fed piglets significantly attenuates both local gastrointestinal and systemic immune-inflammatory responses, with these modulatory effects persisting at least 5 days beyond the treatment period. These results highlight the intricate interaction between gastrointestinal health and systemic immune function in the context of early-life AB intervention, providing insights into the underlying mechanistic pathways shaping immune development in neonates.

## Supplementary information

Supplementary Information

## Data Availability

Data will be made available on request.
