# Supplementary material for: Neonatal enteral antibiotics reduce gut inflammation and delay systemic immune development in preterm pigs
Source: Pediatr Res. 2025 Oct 3;99(5):1945–53. doi: 10.1038/s41390-025-04436-9 (PMC13221306; doi:10.1038/s41390-025-04436-9)
Supplement: Supplementary file 1 — Supplementary Information [file 41390_2025_4436_MOESM1_ESM.pdf]

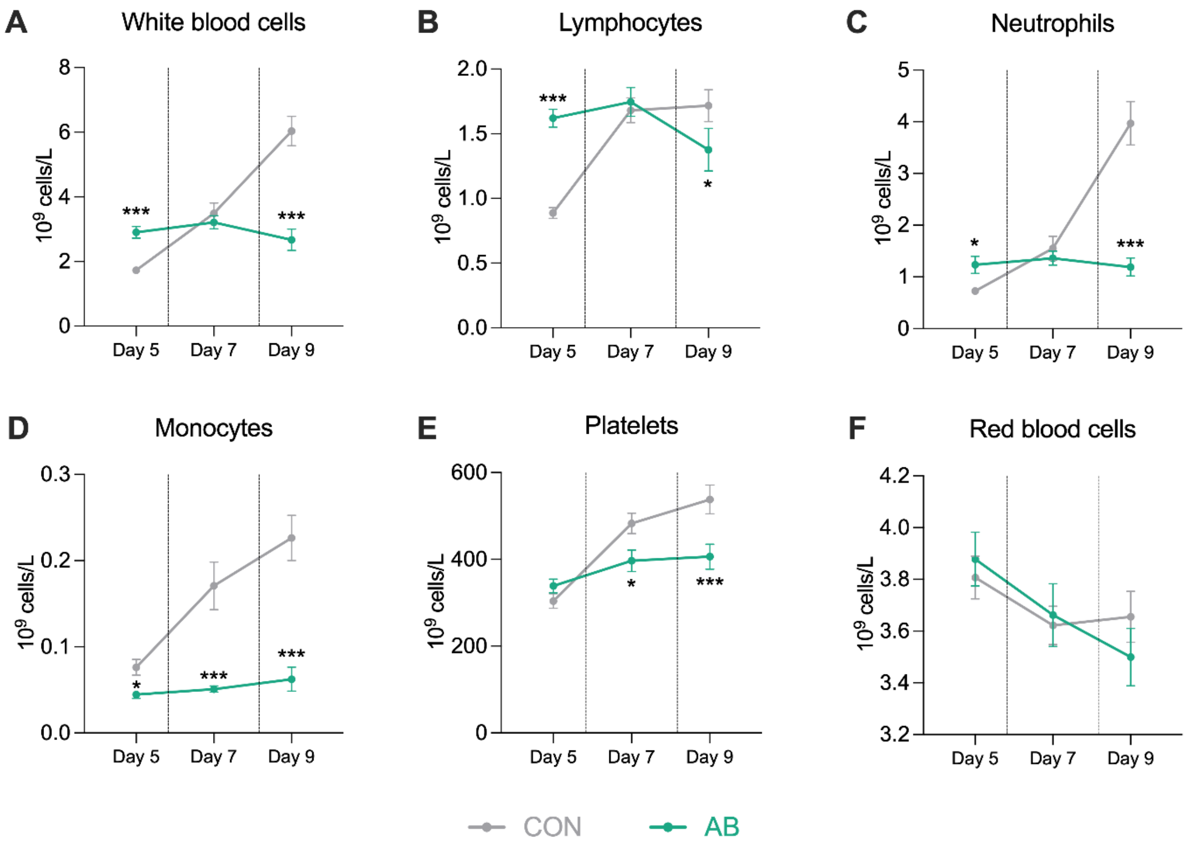

Figure S1. Blood cell count. (A–F) White blood cells, lymphocytes, neutrophils, monocytes, platelets, and red blood cells counts. Data at each time point were analyzed using a linear mixed-effects model, incorporating group, FMT intervention, litter, sex, and birth weight. Values are presented as means ± SEM. *P-value < 0.05, **P-value < 0.01, and ***P-value < 0.001, compared between CON control and AB antibiotics treatment at the same time point.

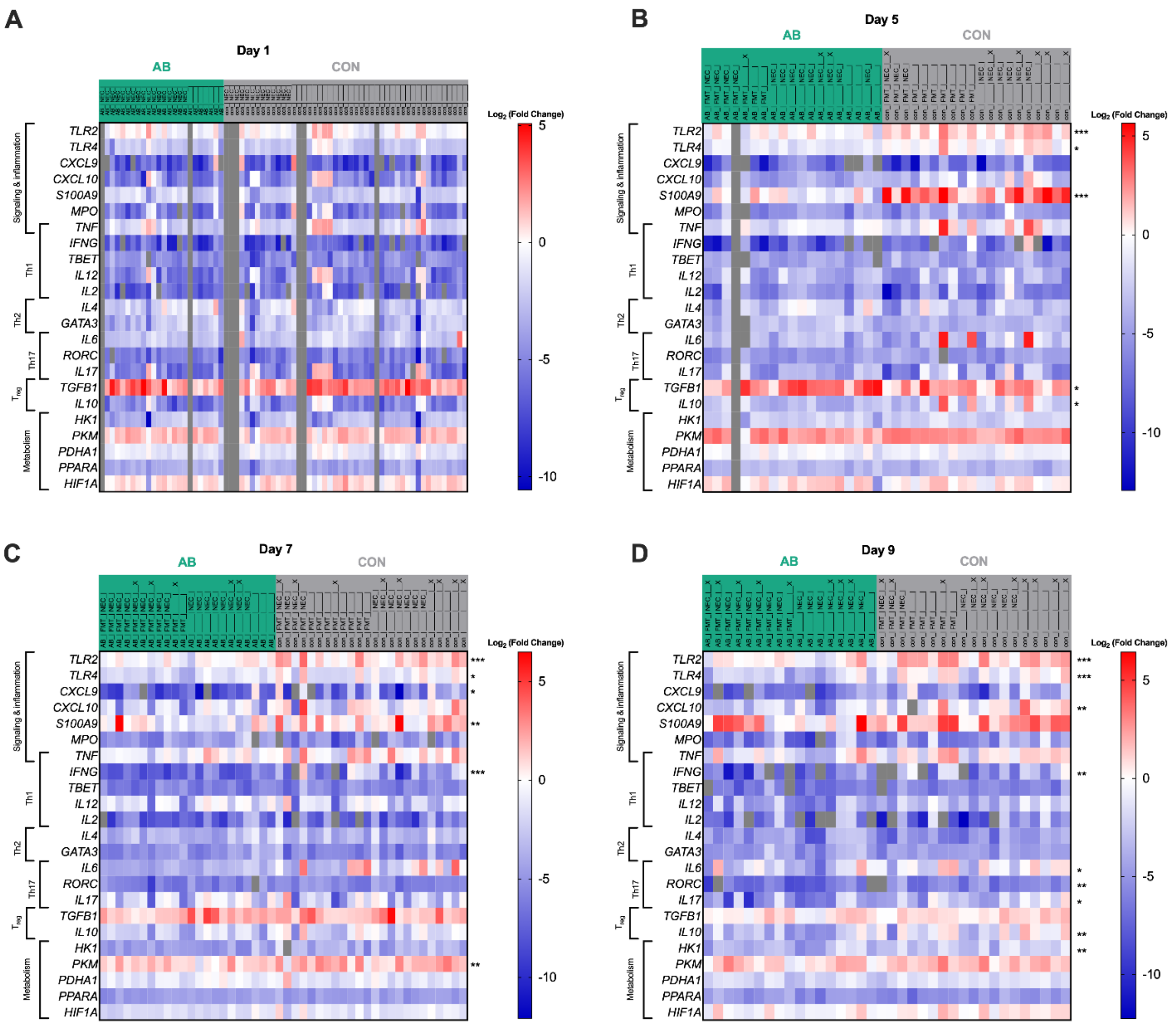

Figure S2. Broad-spectrum antibiotic treatment reduces expression of immune and inflammatory genes in the blood. (A–D) Whole blood gene expressions related to immune functions on day 1, 5, 7, and 9 between AB and CON animals are shown as relative fold changes in relation to HPRT1 expression. The heatmap color palette represents the lowest fold change with cool colors, while warm colors represent the highest values. CON, control; AB, antibiotics; HPRT1, hypoxanthine phosphoribosyltransferase. Relative gene expression in the whole blood by reverse transcription quantitative real-time PCR. $*$ $P < 0.05$, $**$ $P < 0.01$, $***$ $P < 0.001$. TLR2, Toll-like receptor 2; TLR4, Toll-like receptor 4; CXCL9, C-X-C motif chemokine ligand 9; CXCL10, C-X-C motif chemokine ligand 10; S100A9, S100 calcium-binding protein A9; MPO, myeloperoxidase; TNF, tumor necrosis factor; IFNG, interferon gamma; TBET, T-box expressed in T cells; IL12, interleukin 12; IL2, interleukin 2; IL4, interleukin 4; GATA3, GATA-binding protein 3; IL6, interleukin 6; RORC, RAR-related orphan receptor C; IL17, interleukin 17; TGFB1, transforming growth factor beta 1; IL10, interleukin 10; HK1, hexokinase 1; PKM, pyruvate kinase M1/2; PDHA1, pyruvate dehydrogenase E1 subunit alpha 1; PPARA, peroxisome proliferator-activated receptor alpha; HIF1A, hypoxia-inducible factor 1-alpha.

Table S1. Clinical and gut outcomes.

Clinical and gut parameters	CON (n = 32)		AB (n = 28)		P value
	N		*N*		
Daily weight gain (day 1-5), g/d	30	17.2 (8.65)	28	22.1 (6.52)	P < 0.01
Daily weight gain (day 5-9), g/d	27	19.3 (14.7)	28	21.7 (13.7)	NS
Daily weight gain (day 1-9), g/d	27	19.1 (10.9)	28	22.9 (13.7)	P = 0.08
Mean NEC score	31	1.66 (0.80)	27	2.04 (0.75)	NS
Days of diarrhea	29	3.83 (1.65)	27	3.59 (1.22)	NS
Gut permeability, L/M ratio	15	27.2 (15.5)	13	11.7 (9.40)	P < 0.001
Small intestine					
CD3 area fraction, %	31	6.43 (3.90)	26	3.61 (1.86)	P < 0.001
Mucin area fraction, %	31	1.65 (0.85)	27	1.50 (0.60)	NS
MPO staining score	31	3.39 (1.31)	26	2.00 (1.30)	P < 0.0001
FISH staining score	31	2.45 (0.77)	27	1.30 (0.54)	P < 0.0001
Sucrase, U/g	31	0.36 (0.16)	27	0.38 (0.17)	NS
Maltase, U/g	31	4.90 (2.85)	27	5.22 (4.71)	NS
Lactase, U/g	31	10.8 (7.14)	27	14.5 (11.0)	P = 0.09
ApN, U/g	31	3.52 (0.94)	27	4.10 (1.62)	P < 0.05
ApA, U/g	31	1.28 (0.39)	27	2.04 (0.90)	P < 0.0001
DDPIV, U/g	31	1.56 (0.78)	27	1.82 (0.84)	NS
IL-8, ng/g	29	74.5 (29.7)	27	75.8 (27.5)	NS
IL-1β, ng/g	29	4.29 (2.87)	27	3.25 (2.64)	NS
TNF-α, ng/g	28	3.08 (2.26)	27	3.01 (1.99)	NS
IL-10, ng/g	29	1.35 (0.27)	27	1.26 (0.22)	P = 0.06
Colon					
CD3 area fraction, %	31	3.31 (3.07)	26	7.04 (11.7)	NS
Musin area fraction, %	31	5.56 (3.01)	26	8.08 (4.54)	P < 0.05
MPO staining score	31	2.94 (2.14)	26	4.04 (2.24)	P = 0.05
FISH staining score	31	1.90 (0.87)	27	1.74 (0.76)	NS
IL-8, ng/g	29	5.97 (4.34)	27	5.91 (5.99)	NS
IL-6, ng/g	29	19.8 (7.43)	27	17.7 (5.85)	NS
TNF-α, ng/g	29	1.41 (0.78)	27	1.03 (0.67)	P < 0.01
Anaerobic bacterial counts, $\log_{10}$ CFU/g	27	3.58 (1.14)	22	2.89 (0.82)	P < 0.01

Data are presented as mean (SD or number). L/M, lactulose/mannitol; MPO, myeloperoxidase; FISH, fluorescence in situ hybridization.

Table S2 Formula milk composition.

**Product**	**Amount (g/l)**	**Company**
SHS seravit, vitamin and mineral mix	12	Nutricia, Allerød, Denmark
SHS Liquigen MCT, lipid emulsion	60	Nutricia
Calogen LCT, lipid emulsion	40	Nutricia
Fantomalt, maltodextrin	30	Nutricia
Variolac, lactose powder	10	Arla Foods Ingredients, Viby, Denmark
Miprodan 40, casein	35	Arla Foods Ingredients
Lacprodan DI-9224, whey protein	30	Arla Foods Ingredients

Table S3 Primer sequences design of qPCR

Symbol	Gene name	5' primer sequence	3' primer sequence	AL
CXCL9	Chemokine (C-X-C motif) ligand 9	GAAAAGCAGTGTTGCCTTGCT	TGATGCAGGAACAACGTCCAT	98
CXCL10	Chemokine (C-X-C motif) ligand 10	ATCATCCCGAGCTGTTGAGC	CCAGGACTTGGCACATTCAC	94
GATA3	GATA3	ACCCCTTATTAAGCCCAAGC	TCCAGAGAGTCGTCGTTGTG	92
HIF1A	Hypoxia-inducible factor 1-alpha	TGTGTTATCTGTCGCTTTGAGTC	TTTCGCTTTCTCTGAGCATTC	96
HK1	Hexokinase-1	TTTCCCTTGTCGGCAATCCA	CCTCCACTCCGCTTGCTTTA	80
*HPRT1**	Hypoxanthine-guanine phosphoribosyltransferase	TATGGACAGGACTGAACGGC	ACACAGAGGGCTACGATGTG	75
IFNG	Interferon γ	AGCTTTGCGTGACTTTGTGT	ATGCTCCTTTGAATGGCCTG	247
IL2	Interleukin 2	AAGCTCTGGAGGGAGTGCTA	CAACAGCAGTTACTGTCTCATCA	159
IL4	Interleukin 4	GTACCAGCAACTTCGTCCAC	CCTTCTCCGTCGTGTTCTCT	150
IL6	Interleukin 6	TGCCACCTCAGACAAAATGC	AGGTTCAGGTTGTTTTCTGCC	159
IL10	Interleukin 10	GTCCGACTCAACGAAGAAGG	GCCAGGAAGATCAGGCAATA	73
IL12A	Interleukin 12	TCCTGGGAAAGTCCTGTCGT	GGTGAGGTCGCTAGTTTGGA	81
IL17A	Interleukin 17	GCACACGGGCTGCATCAACG	TGCAACCAACAGTGACCCGCA	149
MPO	Myeloperoxidase	CCCGAGTTGCTTTCCTCACT	AAGAAGGGGATGCAGTCACG	127
PDHA1	Pyruvate dehydrogenase E1 α	GTCAGGAAGCTTGTTGCGTG	GGTAAAGCCATGAGCTCGGT	86
PKM	Pyruvate kinase	GCCCTGGACACTAAAGGACC	CAGCCACAGGACATTCTCGT	147
PPARA	Peroxisome proliferator-activated receptor alpha	CCGAGACCGCAGATCTCAAG	GACGAAAGGCGGGTTATTGC	128
RORC	RAR-related orphan receptor γ	CAGCGCTCCAACATCTTCTC	GACCAGCACCACTTCCATTG	207
S100A9	S100-A9	GCCAAACTTTCTCAAGAAGCA	AGTGTCCAGGTCTTCCAGGAT	70
TBX21	T-box TBX21 (T-bet)	CTGAGAGTCGCGCTCAACAA	ACCCGGCCACAGTAAATGAC	121
TGFB1	Transforming growth factor beta-1	GCAAGGTCCTGGCTCTGTA	TAGTACACGATGGGCAGTGG	97
TLR2	Toll-like receptor 2	CGTGTGCTATGACGCTTTCG	GTACTTGCACCACTCGCTCT	232
TLR4	Toll-like receptor 4	TGGTGTCCCAGCACTTCATA	CAACTTCTGCAGGACGATGA	116
TNF	Tumor necrosis factor α	ATTCAGGGATGTGTGGCCTG	CCAGATGTCCCAGGTTGCAT	120

Genes are ordered alphabetically according to gene symbol. AL, amplicon length. *, reference gene.

Table S4. Clinical and gut outcomes in four groups and statistical analyses summary.

Clinical and gut parameters	CON-CON (n = 16)		CON-FMT (n = 16)		AB-CON (n = 13)		AB-FMT (n = 15)		P value (AB)	P value (FMT)	P value (Int.)
	N		***N***		***N***		***N***				
Daily weight gain (day 1-5), g/d	14	19.0 (6.10)	16	15.7 (10.3)	13	21.6 (6.81)	15	22.5 (6.47)	P < 0.01	NS	NS
Daily weight gain (day 5-9), g/d	13	17.4 (13.4)	14	21.1 (16.1)	13	19.8 (14.7)	15	23.4 (13.2)	NS	NS	NS
Daily weight gain (day 1-9), g/d	13	19.5 (9.03)	14	18.8 (12.7)	13	21.7 (10.5)	15	24.0 (8.60)	P = 0.08	NS	NS
Mean NEC score	15	2.02 (0.95)	16	1.33 (0.45)	12	1.85 (0.63)	15	2.18 (0.82)	NS	NS	NS
Days of diarrhea	13	4.15 (1.72)	16	3.56 (1.59)	12	3.50 (1.38)	15	3.67 (1.11)	NS	NS	NS
Gut permeability, L/M ratio	6	24.3 (14.1)	9	29.1 (16.9)	4	8.12 (11.0)	9	13.4 (8.82)	P < 0.001	NS	NS
Small intestine											
CD3 area fraction, %	15	5.63 (2.64)	16	7.17 (4.75)	11	3.41 (1.55)	15	3.76 (2.10)	P < 0.001	NS	NS
Musin area fraction, %	15	1.47 (0.66)	16	1.83 (0.98)	12	1.40 (0.58)	15	1.58 (0.64)	NS	NS	NS
MPO staining score	15	3.60 (1.55)	16	3.19 (1.05)	11	2.18 (1.72)	15	1.87 (0.92)	P < 0.0001	NS	NS
FISH staining score	15	2.67 (0.49)	16	2.25 (0.93)	12	1.08 (0.51)	15	1.47 (0.52)	P < 0.0001	NS	P < 0.05
Sucrase, U/g	15	0.36 (0.21)	16	0.36 (0.10)	12	0.41 (0.23)	15	0.35 (0.10)	NS	NS	NS
Maltase, U/g	15	5.08 (3.03)	16	4.73 (2.75)	12	4.74 (3.71)	15	5.60 (5.48)	NS	NS	NS
Lactase, U/g	15	11.0 (9.08)	16	10.7 (5.00)	12	14.0 (12.2)	15	15.0 (10.3)	P = 0.09	NS	NS
ApN, U/g	15	3.40 (1.03)	16	3.63 (0.86)	12	4.07 (1.74)	15	4.13 (1.57)	P < 0.05	NS	NS
ApA, U/g	15	1.23 (0.36)	16	1.33 (0.42)	12	2.00 (0.91)	15	2.07 (0.92)	P < 0.0001	NS	NS
DDPIV, U/g	15	1.56 (0.97)	16	1.55 (0.57)	12	1.68 (0.84)	15	1.92 (0.86)	NS	NS	NS
IL-8, ng/g	13	69.9 (23.6)	16	78.1 (34.2)	12	70.0 (26.1)	15	80.4 (28.6)	NS	NS	NS
IL-1β, ng/g	13	4.19 (2.32)	16	4.37 (3.32)	12	3.93 (3.57)	15	2.72 (1.49)	NS	NS	NS
TNF-α, ng/g	12	2.94 (2.39)	16	3.19 (2.23)	12	2.01 (1.25)	15	3.81 (2.14)	NS	NS	NS
IL-10, ng/g	13	1.28 (0.21)	16	1.40 (0.31)	12	1.25 (0.20)	15	1.26 (0.24)	P = 0.06	NS	NS
Colon											
CD3 area fraction, %	15	3.00 (2.51)	16	3.60 (3.58)	11	7.10 (14.6)	15	7.00 (9.50)	NS	NS	NS
Musin area fraction, %	15	5.45 (3.35)	16	5.66 (2.75)	11	8.14 (4.88)	15	8.04 (4.44)	P < 0.05	NS	NS

MPO staining score	15	3.60 (2.50)	16	2.31 (1.58)	11	3.36 (2.34)	15	4.53 (2.10)	$P = 0.05$	NS	$P < 0.05$
FISH staining score	15	1.93 (1.03)	16	1.88 (0.72)	12	1.83 (0.83)	15	1.67 (0.72)	NS	NS	NS
IL-8, ng/g	13	5.14 (4.72)	16	6.65 (4.02)	12	8.94 (7.92)	15	3.48 (1.76)	NS	NS	$P < 0.01$
IL-6, ng/g	13	15.5 (6.32)	16	23.3 (6.46)	12	17.2 (5.29)	15	18.1 (6.43)	NS	$P < 0.01$	$P < 0.01$
TNF-α, ng/g	13	1.19 (0.84)	16	1.60 (0.69)	12	0.91 (0.66)	15	1.13 (0.68)	$P < 0.01$	NS	NS
Anaerobic bacterial counts, $\log_{10}$ CFU/g	15	3.76 (1.33)	14	3.40 (0.96)	10	2.62 (0.48)	12	3.11 (0.98)	$P < 0.01$	NS	$P = 0.08$

Data are presented as mean (SD or number). L/M, lactulose/mannitol; MPO, myeloperoxidase; FISH, fluorescence in situ hybridization.

Table S5 KEGG-based gene set enrichment analysis results

ID	Description	setSize	enrichmentSc	NES	pvalue	p.adjust	qvalue	rank	Count	GeneRatio	Sign
ssc03010	Ribosome	106	0.75147863	3.40605701	1.3659E-30	4.453E-28	2.5881E-28	1229	79	0.74528302	Activated
ssc00190	Oxidative phosphorylation	104	0.49788432	2.25554975	2.2475E-08	4.3098E-07	2.5049E-07	1058	43	0.41346154	Activated
ssc00860	Porphyrin metabolism	23	0.66384178	2.11974188	7.6195E-05	0.00048705	0.00028308	1346	10	0.43478261	Activated
ssc03040	Spliceosome	113	0.4478227	2.0652584	1.4262E-06	1.6605E-05	9.6512E-06	1987	42	0.37168142	Activated
ssc04714	Thermogenesis	174	0.41894129	2.02471163	1.6546E-08	3.3713E-07	1.9594E-07	1418	58	0.33333333	Activated
ssc04260	Cardiac muscle contraction	41	0.53705644	1.98852747	5.2632E-05	0.00036506	0.00021218	718	13	0.31707317	Activated
ssc00982	Drug metabolism - cytochrome P450	17	0.64502552	1.90328545	0.00127434	0.00525866	0.00305637	2074	10	0.58823529	Activated
ssc05415	Diabetic cardiomyopathy	154	0.38044822	1.83250879	9.6245E-06	7.844E-05	4.559E-05	1684	56	0.36363636	Activated
ssc05012	Parkinson disease	201	0.36812641	1.8148678	9.6176E-07	1.2541E-05	7.2892E-06	1618	70	0.34825871	Activated
ssc05016	Huntington disease	221	0.36187777	1.80705537	6.5631E-07	9.7253E-06	5.6524E-06	1618	76	0.3438914	Activated
ssc04723	Retrograde endocannabinoid signaling	81	0.4056185	1.7191951	0.00040424	0.00213286	0.00123963	1418	27	0.33333333	Activated
ssc00280	Valine, leucine and isoleucine degradation	40	0.46993522	1.71884481	0.00400102	0.0140251	0.00815149	2655	20	0.5	Activated
ssc05014	Amyotrophic lateral sclerosis	260	0.32543804	1.6478035	8.6904E-06	7.4555E-05	4.3332E-05	1677	79	0.30384615	Activated
ssc03030	DNA replication	30	0.48078105	1.6412424	0.00856193	0.02584434	0.01502093	2385	13	0.43333333	Activated
ssc05020	Prion disease	191	0.334129	1.62604102	8.4644E-05	0.00053065	0.00030842	1618	64	0.33507853	Activated
ssc04932	Non-alcoholic fatty liver disease	121	0.35168215	1.59624225	0.00090445	0.00398447	0.0023158	1058	34	0.28099174	Activated
ssc03020	RNA polymerase	30	0.46323101	1.58133179	0.01747521	0.04747431	0.02759243	2016	14	0.46666667	Activated
ssc03420	Nucleotide excision repair	53	0.39863223	1.54955287	0.00812936	0.02476797	0.01439533	2385	22	0.41509434	Activated
ssc03008	Ribosome biogenesis in eukaryotes	68	0.37875804	1.54081799	0.00293078	0.010982	0.00638282	3127	30	0.44117647	Activated
ssc05171	Coronavirus disease - COVID-19	160	0.32111441	1.53615241	0.00048291	0.00249889	0.00145237	910	53	0.33125	Activated
ssc05208	Chemical carcinogenesis - reactive oxygen species	169	0.3042875	1.44668015	0.00238142	0.00913344	0.00530842	1001	47	0.27810651	Activated
ssc05022	Pathways of neurodegeneration - multiple diseases	317	0.26253473	1.36208671	0.00071214	0.00340701	0.00198018	1559	85	0.2681388	Activated
ssc05010	Alzheimer disease	270	0.25776549	1.30898695	0.00913218	0.02731275	0.01587438	1553	69	0.25555556	Activated
ssc04810	Regulation of actin cytoskeleton	141	-0.4048959	-1.457232	0.00579223	0.01907339	0.0110856	2098	46	0.32624113	Suppressed
ssc04910	Insulin signaling pathway	109	-0.4165146	-1.4700747	0.01630991	0.04468093	0.0259689	1986	33	0.30275229	Suppressed
ssc05166	Human T-cell leukemia virus 1 infection	178	-0.3992685	-1.4737995	0.00472172	0.01603417	0.00931918	2042	58	0.3258427	Suppressed
ssc04072	Phospholipase D signaling pathway	95	-0.4346298	-1.5100251	0.01131561	0.03180076	0.01848285	3223	49	0.51578947	Suppressed
ssc04722	Neurotrophin signaling pathway	90	-0.4431003	-1.5214796	0.00697017	0.02164071	0.01257775	2387	42	0.46666667	Suppressed
ssc04919	Thyroid hormone signaling pathway	90	-0.4451385	-1.5284783	0.00609343	0.01947508	0.01131907	2520	39	0.43333333	Suppressed
ssc05211	Renal cell carcinoma	58	-0.4810107	-1.5364683	0.01052379	0.03041394	0.01767681	2050	26	0.44827586	Suppressed
ssc05224	Breast cancer	79	-0.4600536	-1.5458375	0.00802654	0.02468539	0.01434733	1952	30	0.37974684	Suppressed
ssc05215	Prostate cancer	71	-0.4722705	-1.5593743	0.01054225	0.03041394	0.01767681	2520	32	0.45070423	Suppressed
ssc05170	Human immunodeficiency virus 1 infection	153	-0.4304588	-1.5597248	0.00122912	0.0051371	0.00298572	2186	56	0.36601307	Suppressed
ssc04520	Adherens junction	71	-0.472716	-1.5608454	0.01035892	0.03041394	0.01767681	2560	34	0.47887324	Suppressed
ssc03250	Viral life cycle - HIV-1	47	-0.5100046	-1.5725565	0.00945141	0.02801055	0.01627994	2042	16	0.34042553	Suppressed
ssc04611	Platelet activation	89	-0.4610078	-1.5781203	0.0057631	0.01907339	0.0110856	2387	35	0.39325843	Suppressed
ssc05222	Small cell lung cancer	64	-0.4875664	-1.5908433	0.00672947	0.02109431	0.01226018	2259	28	0.4375	Suppressed
ssc05132	Salmonella infection	188	-0.4294758	-1.5955449	0.00054526	0.00277742	0.00161426	2540	78	0.41489362	Suppressed
ssc04917	Prolactin signaling pathway	48	-0.5150684	-1.5979397	0.01089668	0.03115775	0.01810912	2479	30	0.625	Suppressed
ssc05212	Pancreatic cancer	67	-0.4862191	-1.5984492	0.00596767	0.01926197	0.0111952	2050	29	0.43283582	Suppressed
ssc04610	Complement and coagulation cascades	34	-0.5564034	-1.6221731	0.01348093	0.03756224	0.02183146	924	13	0.38235294	Suppressed
ssc00052	Galactose metabolism	24	-0.603557	-1.6305041	0.01099123	0.03115775	0.01810912	1018	9	0.375	Suppressed
ssc04071	Sphingolipid signaling pathway	90	-0.4759686	-1.63434	0.00185568	0.00728858	0.00423618	2832	43	0.47777778	Suppressed
ssc04612	Antigen processing and presentation	42	-0.5436152	-1.6349512	0.00592901	0.01926197	0.0111952	1836	16	0.38095238	Suppressed
ssc00500	Starch and sucrose metabolism	22	-0.6157952	-1.6370993	0.01401905	0.0387306	0.02251052	974	8	0.36363636	Suppressed
ssc05146	Amoebiasis	61	-0.5113306	-1.6486347	0.00285066	0.01080597	0.00628051	2368	31	0.50819672	Suppressed
ssc05168	Herpes simplex virus 1 infection	232	-0.4363783	-1.6514484	5.5428E-05	0.00037645	0.00021879	1968	62	0.26724138	Suppressed
ssc04670	Leukocyte transendothelial migration	76	-0.494167	-1.6533883	0.00150399	0.00612875	0.00356208	2390	34	0.44736842	Suppressed
ssc04928	Parathyroid hormone synthesis, secretion and action	74	-0.4979214	-1.6651987	0.00153924	0.00619498	0.00360057	1944	28	0.37837838	Suppressed
ssc04724	Glutamatergic synapse	55	-0.5243754	-1.6696221	0.00396638	0.0140251	0.00815149	3018	31	0.56363636	Suppressed
ssc04726	Serotonergic synapse	57	-0.5230605	-1.6697165	0.00391448	0.01402329	0.00815044	3018	34	0.59649123	Suppressed
ssc04935	Growth hormone synthesis, secretion and action	79	-0.497914	-1.6730531	0.00101069	0.00439313	0.00255332	3040	44	0.55696203	Suppressed
ssc05205	Proteoglycans in cancer	134	-0.4708842	-1.6804575	0.00040564	0.00213286	0.00123963	2520	57	0.42537313	Suppressed
ssc05144	Malaria	35	-0.5736818	-1.6820667	0.00508669	0.01709546	0.00993601	2393	21	0.6	Suppressed
ssc04024	cAMP signaling pathway	105	-0.4799622	-1.6830837	0.0008779	0.00392049	0.00227862	2355	42	0.4	Suppressed
ssc05143	African trypanosomiasis	21	-0.6406807	-1.6847468	0.00661538	0.020938	0.01216932	2324	14	0.66666667	Suppressed
ssc04912	GnRH signaling pathway	62	-0.5224573	-1.6890325	0.00316324	0.01158669	0.00673427	1952	25	0.40322581	Suppressed
ssc04142	Lysosome	112	-0.4786463	-1.6917	0.00065188	0.0032199	0.00187143	1859	50	0.44642857	Suppressed
ssc04144	Endocytosis	196	-0.4547744	-1.6929381	5.8252E-05	0.00038254	0.00022233	2584	75	0.38265306	Suppressed
ssc04930	Type II diabetes mellitus	28	-0.6093115	-1.6932756	0.00468766	0.01603417	0.00931918	1854	12	0.42857143	Suppressed
ssc05134	Legionellosis	44	-0.5608579	-1.7047135	0.00464783	0.01603417	0.00931918	2368	19	0.43181818	Suppressed
ssc04015	Rap1 signaling pathway	120	-0.4808575	-1.7120982	0.0002419	0.00133659	0.00077683	3040	63	0.525	Suppressed
ssc04933	AGE-RAGE signaling pathway in diabetic complications	72	-0.5157567	-1.7137551	0.00164164	0.00652654	0.00379327	2560	36	0.5	Suppressed
ssc04666	Fc gamma R-mediated phagocytosis	78	-0.5120698	-1.7149036	0.00073811	0.00340701	0.00198018	2295	35	0.44871795	Suppressed
ssc05207	Chemical carcinogenesis - receptor activation	103	-0.4921178	-1.7176427	0.00065183	0.0032199	0.00187143	1992	40	0.38834951	Suppressed
ssc04210	Apoptosis	102	-0.4928066	-1.719878	0.00074122	0.00340701	0.00198018	1854	41	0.40196078	Suppressed
ssc05206	MicroRNAs in cancer	126	-0.484033	-1.7232084	0.00024044	0.00133659	0.00077683	2108	48	0.38095238	Suppressed
ssc02010	ABC transporters	23	-0.6462655	-1.7241611	0.00382662	0.01386086	0.00805604	1096	8	0.34782609	Suppressed
ssc04623	Cytosolic DNA-sensing pathway	56	-0.5406399	-1.7252823	0.0022395	0.00869139	0.0050515	1416	18	0.32142857	Suppressed
ssc05203	Viral carcinogenesis	142	-0.480497	-1.7308795	9.6486E-05	0.00059348	0.00034493	2050	51	0.35915493	Suppressed
ssc00600	Sphingolipid metabolism	38	-0.5845142	-1.7371322	0.00315247	0.01158669	0.00673427	1763	14	0.36842105	Suppressed
ssc05200	Pathways in cancer	328	-0.4544007	-1.7412431	2.8629E-07	4.9121E-06	2.8549E-06	2267	129	0.39329268	Suppressed
ssc04662	B cell receptor signaling pathway	71	-0.5289898	-1.7466539	0.00111791	0.00473298	0.00275084	2148	34	0.47887324	Suppressed
ssc05235	PD-L1 expression and PD-1 checkpoint pathway in cancer	75	-0.5220237	-1.7472895	0.00069037	0.0033591	0.00195233	2715	40	0.53333333	Suppressed
ssc04510	Focal adhesion	123	-0.4924339	-1.7535763	0.00010582	0.00062724	0.00036455	2504	52	0.42276423	Suppressed
ssc04650	Natural killer cell mediated cytotoxicity	73	-0.5256575	-1.7562039	0.00074202	0.00340701	0.00198018	2113	36	0.49315068	Suppressed
ssc04512	ECM-receptor interaction	42	-0.5888523	-1.7710044	0.00108617	0.0046591	0.0027079	1800	16	0.38095238	Suppressed
ssc04920	Adipocytokine signaling pathway	47	-0.5766985	-1.7782014	0.00079014	0.00357759	0.00207932	1581	18	0.38297872	Suppressed
ssc04151	PI3K-Akt signaling pathway	188	-0.4788311	-1.7789046	6.9562E-06	6.6697E-05	3.8765E-05	2335	66	0.35106383	Suppressed
ssc04014	Ras signaling pathway	132	-0.500126	-1.7827076	3.2993E-05	0.00023382	0.0001359	2347	55	0.41666667	Suppressed
ssc04066	HIF-1 signaling pathway	79	-0.5312487	-1.7850619	0.00014629	0.00085162	0.00049497	2520	41	0.51898734	Suppressed
ssc05163	Human cytomegalovirus infection	162	-0.4905079	-1.7861645	1.3726E-05	0.00010914	6.3431E-05	3059	84	0.51851852	Suppressed
ssc04514	Cell adhesion molecules	79	-0.5352641	-1.798554	9.9995E-05	0.00060367	0.00035086	1770	27	0.34177215	Suppressed
ssc04217	Necroptosis	98	-0.5174206	-1.8014577	0.00020502	0.00117255	0.00068149	1890	38	0.3877551	Suppressed
ssc04010	MAPK signaling pathway	180	-0.4980048	-1.8445355	7.793E-07	1.093E-05	6.3526E-06	2598	83	0.46111111	Suppressed
ssc04145	Phagosome	96	-0.5367397	-1.8701313	1.9737E-05	0.00014624	8.4993E-05	2183	43	0.44791667	Suppressed
ssc04062	Chemokine signaling pathway	130	-0.5259384	-1.8721338	9.1623E-06	7.6588E-05	4.4513E-05	2185	55	0.42307692	Suppressed
ssc05150	Staphylococcus aureus infection	28	-0.681964	-1.8951767	0.00026187	0.00142282	0.00082696	512	9	0.32142857	Suppressed
ssc05165	Human papillomavirus infection	201	-0.5074556	-1.8993345	2.0088E-07	3.6382E-06	2.1145E-06	1952	74	0.3681592	Suppressed
ssc05135	Yersinia infection	117	-0.5360508	-1.9053778	6.3509E-06	6.2739E-05	3.6464E-05	2289	49	0.41880342	Suppressed
ssc04936	Alcoholic liver disease	93	-0.5573289	-1.9255419	1.8166E-05	0.00013773	8.0048E-05	2390	41	0.44086022	Suppressed
ssc04659	Th17 cell differentiation	87	-0.5648275	-1.9284261	1.627E-05	0.00012628	7.3398E-05	1543	42	0.48275862	Suppressed
ssc04657	IL-17 signaling pathway	57	-0.6106475	-1.9493125	5.8671E-05	0.00038254	0.00022233	2387	30	0.52631579	Suppressed
ssc05202	Transcriptional misregulation in cancer	125	-0.549763	-1.9603446	8.0467E-07	1.093E-05	6.3526E-06	1355	41	0.328	Suppressed
ssc04658	Th1 and Th2 cell differentiation	69	-0.596371	-1.9651983	2.4745E-05	0.00017926	0.00010419	1875	31	0.44927536	Suppressed
ssc05161	Hepatitis B	130	-0.557103	-1.9830674	3.9396E-07	6.4216E-06	3.7323E-06	1953	58	0.44615385	Suppressed
ssc04625	C-type lectin receptor signaling pathway	80	-0.5931404	-1.993691	6.0984E-06	6.2127E-05	3.6109E-05	2289	42	0.525	Suppressed
ssc04064	NF-kappa B signaling pathway	78	-0.5985707	-2.0045918	2.8552E-06	3.0025E-05	1.7451E-05	2368	44	0.56410256	Suppressed
ssc05142	Chagas disease	79	-0.5974309	-2.0074422	1.0629E-06	1.3327E-05	7.7457E-06	1944	35	0.44303797	Suppressed
ssc05140	Leishmaniasis	56	-0.6349364	-2.0262	8.0614E-06	7.384E-05	4.2916E-05	2387	35	0.625	Suppressed
ssc04622	RIG-I-like receptor signaling pathway	50	-0.6524883	-2.0367213	8.1541E-06	7.384E-05	4.2916E-05	1697	24	0.48	Suppressed
ssc05323	Rheumatoid arthritis	57	-0.6415584	-2.0479864	8.5402E-06	7.4555E-05	4.3332E-05	1914	28	0.49122807	Suppressed
ssc05167	Kaposi sarcoma-associated herpesvirus infection	139	-0.5737523	-2.0568936	5.2476E-09	1.5552E-07	9.0389E-08	2148	68	0.48920863	Suppressed
ssc05169	Epstein-Barr virus infection	158	-0.5671195	-2.0629353	1.0901E-09	5.495E-08	3.1938E-08	2050	64	0.40506329	Suppressed
ssc05133	Pertussis	56	-0.6521718	-2.0812014	2.1035E-06	2.3646E-05	1.3743E-05	2387	33	0.58928571	Suppressed
ssc05145	Toxoplasmosis	81	-0.6190447	-2.0837019	4.1488E-07	6.4406E-06	3.7433E-06	1944	38	0.4691358	Suppressed
ssc05152	Tuberculosis	123	-0.5863717	-2.0880926	1.4438E-08	3.3609E-07	1.9534E-07	2431	64	0.5203252	Suppressed
ssc04060	Cytokine-cytokine receptor interaction	121	-0.5868884	-2.0927972	1.5464E-08	3.3609E-07	1.9534E-07	1095	51	0.4214876	Suppressed
ssc05321	Inflammatory bowel disease	46	-0.6824547	-2.0936994	1.4143E-06	1.6605E-05	9.6512E-06	1774	24	0.52173913	Suppressed
ssc05417	Lipid and atherosclerosis	155	-0.5796244	-2.1043413	6.4935E-10	5.2922E-08	3.0759E-08	2387	76	0.49032258	Suppressed
ssc04061	Viral protein interaction with cytokine and cytokine receptor	48	-0.6786671	-2.1054859	2.2143E-06	2.4062E-05	1.3985E-05	1095	19	0.39583333	Suppressed
ssc04621	NOD-like receptor signaling pathway	117	-0.5947698	-2.1140927	1.3613E-08	3.3609E-07	1.9534E-07	1890	50	0.42735043	Suppressed
ssc04148	Efferocytosis	121	-0.6033358	-2.1514474	1.4613E-09	5.495E-08	3.1938E-08	2108	57	0.47107438	Suppressed
ssc04630	JAK-STAT signaling pathway	92	-0.63347	-2.178907	5.9689E-09	1.6216E-07	9.4246E-08	1890	47	0.51086957	Suppressed
ssc05160	Hepatitis C	104	-0.6266641	-2.1907582	1.517E-09	5.495E-08	3.1938E-08	1952	53	0.50961538	Suppressed
ssc04380	Osteoclast differentiation	107	-0.6217985	-2.1909927	1.6958E-09	5.5283E-08	3.2131E-08	2289	61	0.57009346	Suppressed
ssc05162	Measles	107	-0.6248016	-2.2015744	9.522E-10	5.495E-08	3.1938E-08	1607	44	0.41121495	Suppressed
ssc04620	Toll-like receptor signaling pathway	79	-0.6582465	-2.2117903	1.4485E-09	5.495E-08	3.1938E-08	2387	48	0.60759494	Suppressed
ssc04668	TNF signaling pathway	90	-0.6805378	-2.336772	1.2387E-11	1.3461E-09	7.8235E-10	1882	49	0.54444444	Suppressed
ssc05164	Influenza A	122	-0.6580489	-2.346399	3.5378E-13	5.7666E-11	3.3516E-11	1912	64	0.52459016	Suppressed

Table S6 KEGG-based gene set variation analysis results

Pathway	logFC	AveExpr	t	P.Value	adj.P.Val	B
KEGG_TYPE_II_DIABETES_MELLITUS	-0.4629145	0.01219419	-5.0841102	2.76E-05	0.00514124	2.54834748
KEGG_SELENOAMINO_ACID_METABOLISM	0.39456711	0.02160185	4.52406503	0.00012041	0.01119822	1.20902136
KEGG_B_CELL_RECEPTOR_SIGNALING_PATHWAY	-0.4016425	-0.0300069	-4.0398689	0.0004275	0.02618156	0.05569786
KEGG_PANCREATIC_CANCER	-0.3607361	-0.0065094	-3.9201192	0.00058337	0.02618156	-0.2268142
KEGG_DORSO_VENTRAL_AXIS_FORMATION	-0.4868804	0.00165202	-3.8030456	0.00078944	0.02618156	-0.501395
KEGG_ACUTE_MYELOID_LEUKEMIA	-0.3549478	-0.014694	-3.7482763	0.00090894	0.02618156	-0.6292141
KEGG_INSULIN_SIGNALING_PATHWAY	-0.2910353	-0.0198155	-3.716855	0.00098533	0.02618156	-0.7023436
KEGG_TOLL_LIKE_RECEPTOR_SIGNALING_PATHWAY	-0.4519464	-0.120129	-3.6016968	0.00132282	0.02742421	-0.9689961
KEGG_CHRONIC_MYELOID_LEUKEMIA	-0.3525162	-0.0183723	-3.565998	0.0014487	0.02742421	-1.0511887
KEGG_ENDOCYTOSIS	-0.2668999	-0.0522379	-3.5144871	0.00165114	0.02742421	-1.1693633
KEGG_LEISHMANIA_INFECTION	-0.4144736	-0.0732186	-3.4630413	0.00188071	0.02742421	-1.2868628
KEGG_RIG_I_LIKE_RECEPTOR_SIGNALING_PATHWAY	-0.3880277	-0.1672857	-3.4432275	0.00197716	0.02742421	-1.3319699
KEGG_SULFUR_METABOLISM	0.31124691	-0.2622357	3.38560026	0.00228576	0.02742421	-1.4626767
KEGG_PROSTATE_CANCER	-0.2937985	-0.018218	-3.3603354	0.00243534	0.02742421	-1.5197454
KEGG_ABC_TRANSPORTERS	-0.3066189	0.00482284	-3.3595168	0.00244034	0.02742421	-1.521592
KEGG_NATURAL_KILLER_CELL_MEDIATED_CYTOTOXICITY	-0.2903251	-0.2121495	-3.3535274	0.00247724	0.02742421	-1.5350983
KEGG_EPITHELIAL_CELL_SIGNALING_IN_HELICOBACTER_PYLORI_INFECTION	-0.4142142	-0.0312562	-3.3212556	0.00268553	0.02742421	-1.607725
KEGG_JAK_STAT_SIGNALING_PATHWAY	-0.4135423	-0.1084919	-3.3133276	0.00273924	0.02742421	-1.6255285
KEGG_NEUROTROPHIN_SIGNALING_PATHWAY	-0.3049182	-0.0285013	-3.2986601	0.00284135	0.02742421	-1.658426
KEGG_CHEMOKINE_SIGNALING_PATHWAY	-0.3301665	-0.0591859	-3.28376	0.00294884	0.02742421	-1.6917904
KEGG_APOPTOSIS	-0.3548234	-0.0681083	-3.2234791	0.00342515	0.03033705	-1.826195
KEGG_GLYCOSAMINOGLYCAN_BIOSYNTHESIS_CHONDROITIN_SULFATE	-0.2981418	-0.0161099	-3.1941976	0.00368247	0.03113365	-1.8911373
KEGG_MTOR_SIGNALING_PATHWAY	-0.2830064	-0.001723	-3.1561212	0.00404501	0.0327118	-1.9752343
KEGG_ALDOSTERONE_REGULATED_SODIUM_REABSORPTION	-0.3437386	-0.0151777	-3.1301375	0.00431183	0.03279861	-2.032389
KEGG_FC_GAMMA_R_MEDIATED_PHAGOCYTOSIS	-0.300665	-0.0426888	-3.1097544	0.00453287	0.03279861	-2.0770881
KEGG_RENAL_CELL_CARCINOMA	-0.3068923	-0.0190511	-3.1051088	0.00458475	0.03279861	-2.0872588
KEGG_VALINE_LEUCINE_AND_ISOLEUCINE_DEGRADATION	0.30992809	0.01008484	3.04293867	0.00533614	0.03676005	-2.2227449
KEGG_ADIPOCYTOKINE_SIGNALING_PATHWAY	-0.3554044	-0.0098996	-3.0158968	0.00569849	0.03773157	-2.2813055
KEGG_SPHINGOLIPID_METABOLISM	-0.2792234	-0.0130345	-3.00276	0.00588288	0.03773157	-2.3096706
KEGG_AUTOIMMUNE_THYROID_DISEASE	-0.2304886	-0.3931572	-2.962545	0.00648337	0.03946509	-2.3961559
KEGG_RIBOSOME	0.66037113	0.05486011	2.956564	0.00657752	0.03946509	-2.408973
KEGG_NON_SMALL_CELL_LUNG_CANCER	-0.2628833	-0.0013576	-2.9050966	0.00744317	0.04159876	-2.5187686
KEGG_ADHERENS_JUNCTION	-0.2585449	-0.0113136	-2.8864959	0.00778183	0.04159876	-2.5582254
KEGG_FOCAL_ADHESION	-0.2699805	-0.0249916	-2.8840768	0.00782694	0.04159876	-2.563348
KEGG_REGULATION_OF_ACTIN_CYTOSKELETON	-0.2347712	-0.0352252	-2.8840348	0.00782772	0.04159876	-2.563437
KEGG_NOD_LIKE_RECEPTOR_SIGNALING_PATHWAY	-0.3908426	-0.0395895	-2.8316285	0.00886793	0.04344668	-2.673898
KEGG_VIRAL_MYOCARDITIS	-0.2367122	-0.1283955	-2.8309994	0.00888117	0.04344668	-2.6752178
KEGG_GLYCOSPHINGOLIPID_BIOSYNTHESIS_GLOBO_SERIES	-0.3143341	-0.040007	-2.8307494	0.00888644	0.04344668	-2.6757424
KEGG_ANTIGEN_PROCESSING_AND_PRESENTATION	-0.2563953	-0.2520856	-2.8109109	0.00931414	0.04344668	-2.7172907
KEGG_PATHWAYS_IN_CANCER	-0.2548103	-0.0098057	-2.8095867	0.00934337	0.04344668	-2.7200588
KEGG_VIBRIO_CHOLERAE_INFECTION	-0.2659309	-0.0209019	-2.7911821	0.0097587	0.04392443	-2.7584641
KEGG_GLYCOSAMINOGLYCAN_DEGRADATION	-0.3437307	-0.0126795	-2.7843007	0.00991842	0.04392443	-2.772791
KEGG_GLIOMA	-0.2451936	-0.0063903	-2.729114	0.01129132	0.0480331	-2.8870304
KEGG_GNRH_SIGNALING_PATHWAY	-0.2550966	-0.0412125	-2.7264219	0.01136267	0.0480331	-2.8925728
KEGG_GLYCOLYSIS_GLUCONEOGENESIS	-0.2672401	-0.0211558	-2.7064294	0.0119059	0.04857879	-2.9336429
KEGG_GALACTOSE_METABOLISM	-0.2789671	-0.0134574	-2.6940609	0.01225409	0.04857879	-2.9589713
KEGG_RIBOFLAVIN_METABOLISM	0.29980676	-0.0174644	2.69331848	0.01227529	0.04857879	-2.9604896
KEGG_ERBB_SIGNALING_PATHWAY	-0.2410664	0.00182387	-2.6649131	0.01311273	0.05053316	-3.0184148
KEGG_CIRCADIAN_RHYTHM_MAMMAL	-0.2981778	-0.0001477	-2.6583885	0.0133125	0.05053316	-3.0316731
KEGG_FC_EPSILON_RI_SIGNALING_PATHWAY	-0.2367202	-0.0195323	-2.6065422	0.01500407	0.05547168	-3.136395
KEGG_MISMATCH_REPAIR	0.29945115	-0.0140589	2.60060819	0.01520998	0.05547168	-3.1483079
KEGG_MAPK_SIGNALING_PATHWAY	-0.2518575	-0.0241578	-2.5115535	0.01863351	0.06606922	-3.3252385
KEGG_BUTANOATE_METABOLISM	0.26966849	0.0075936	2.50700636	0.01882617	0.06606922	-3.3341773
KEGG_LYSINE_DEGRADATION	-0.2121738	-0.0203291	-2.4637061	0.02075537	0.07104711	-3.4188194
KEGG_STARCH_AND_SUCROSE_METABOLISM	-0.263303	-0.0099201	-2.458302	0.02100855	0.07104711	-3.4293218
KEGG_SMALL_CELL_LUNG_CANCER	-0.2552379	-0.028067	-2.433582	0.02220341	0.07374704	-3.477187
KEGG_RNA_POLYMERASE	0.28646835	-0.0395598	2.41151957	0.02332232	0.07610441	-3.5196595
KEGG_CALCIUM_SIGNALING_PATHWAY	-0.1947549	-0.0084725	-2.3841263	0.02478362	0.07947852	-3.5720655
KEGG_LEUKOCYTE_TRANSENDOTHELIAL_MIGRATION	-0.2450675	-0.0238492	-2.3589689	0.02619912	0.08259385	-3.6198684
KEGG_CYTOSOLIC_DNA_SENSING_PATHWAY	-0.2867935	-0.1967884	-2.3505865	0.02668696	0.08272957	-3.6357262
KEGG_LYSOSOME	-0.2529915	-0.0274772	-2.3319602	0.02780081	0.08474415	-3.6708365
KEGG_BLADDER_CANCER	-0.2373404	-0.0198995	-2.3235075	0.02832014	0.08474415	-3.6867116
KEGG_T_CELL_RECEPTOR_SIGNALING_PATHWAY	-0.2257299	-0.0043525	-2.3173556	0.02870367	0.08474415	-3.6982426
KEGG_GRAFT_VERSUS_HOST_DISEASE	-0.2199836	-0.2093599	-2.2803313	0.03111348	0.08953834	-3.7672285
KEGG_PRION_DISEASES	-0.3288919	-0.0175879	-2.268487	0.03192235	0.08953834	-3.7891467
KEGG_CARDIAC_MUSCLE_CONTRACTION	0.29738098	-0.0328835	2.26301652	0.03230235	0.08953834	-3.799245
KEGG_LONG_TERM_DEPRESSION	-0.2274401	-0.039413	-2.2576937	0.03267603	0.08953834	-3.8090556
KEGG_NUCLEOTIDE_EXCISION_REPAIR	0.21083806	-0.012751	2.25686648	0.03273445	0.08953834	-3.8105789
KEGG_PARKINSONS_DISEASE	0.31356451	-0.0341496	2.23974147	0.03396538	0.09155884	-3.8420326
KEGG_HISTIDINE_METABOLISM	0.25681188	0.0045887	2.21865344	0.03553864	0.09443124	-3.8805494
KEGG_DRUG_METABOLISM_CYTOCHROME_P450	0.21913709	-0.2088299	2.18972328	0.03780402	0.09679586	-3.932997
KEGG_ALLOGRAFT_REJECTION	-0.2203342	-0.2032332	-2.1876587	0.03797056	0.09679586	-3.9367224
KEGG_TYROSINE_METABOLISM	0.2643942	0.00192686	2.17656092	0.03887711	0.09679586	-3.9567072
KEGG_NOTCH_SIGNALING_PATHWAY	-0.1858025	-0.0353914	-2.1753387	0.03897813	0.09679586	-3.9589039
KEGG_HUNTINGTONS_DISEASE	0.25793	-0.03008	2.17470512	0.03903059	0.09679586	-3.9600424
KEGG_GLYCOSPHINGOLIPID_BIOSYNTHESIS_GANGLIO_SERIES	-0.2605911	-0.026917	-2.1547766	0.04071341	0.09964072	-3.9957372
